# Comparison of hyaluronic acid-based micelles and polyethylene glycol-based micelles on reversal of multidrug resistance and enhanced anticancer efficacy *in vitro* and *in vivo*

**DOI:** 10.1080/10717544.2018.1428385

**Published:** 2018-01-19

**Authors:** Jinling Wang, Ying Li, Lifang Wang, Xiaohui Wang, Pengfei Tu

**Affiliations:** School of Chinese Materia Medica, Modern Research Center for Traditional Chinese Medicine, Beijing University of Chinese Medicine, Beijing, PR China

**Keywords:** Hyaluronic acid, polyethylene glycol, micelles, multidrug resistance, anticancer effect, co-delivery

## Abstract

Polyethylene glycol (PEG)-based block copolymer micelles and hyaluronic acid (HA)-based grafted copolymer micelles have been widely investigated in chemotherapy. In this study, to evaluate the differences among HA-based grafted polymer micelles, PEG-based block polymer micelles and the mixed of these two micelles in enhancing antitumor effects and overcoming MDR, two amphiphilic vitamin E succinate (VES) derivatives, HA VES (HA-g-VES) and PEG 2000 VES (TPGS2k), were applied as nanocarriers to prepare HA-VES micelles (HA-PMs), TPGS2k micelles (TPGS2k-PMs) and the mixed micelles (HA/TPGS2k-PMs) for the co-delivery of doxorubicin (DOX) and curcumin (Cur). With the addition of TPGS2k, the particle size of HA/TPGS2k-PMs (153.37 ± 1.00 nm) was smaller than that of HA-PMs (223.83 ± 1.84) but significantly larger than that of TPGS2k-PMs (about 20 nm). The loading efficiency of HA/TPGS2k-PMs was 7.10%, which was lower than HA-PMs (8.31 ± 0.15%) but higher than TPGS2k-PMs (4.38 ± 0.24%). *In vitro*, HA/TPGS2k-PMs and TPGS2k-PMs exhibited higher cytotoxicity and reversal MDR effects than HA-PMs in MCF-7/Adr cells. However, HA/TPGS2k-PMs, HA-PMs and TPGS2k-PMs all significantly improved the tumor biodistribution, the antitumor effects and reduced the side effects of DOX in 4T1-tumor-bearing mice, but these three micelles displayed no differences *in vivo*. Therefore, EPR passive targeting effects caused by PEGylated micelles and CD44 active targeting effects caused by HA-based micelles have no significant variance in the delivery of antitumor drugs by i.v.

## 1. Introduction

Chemotherapy plays a critical role in the treatment of cancer. Multidrug resistance (MDR) is a main obstacle in cancer chemotherapy. With recent advances in nanotechnology, due to the unique characteristics such as the nano-size, a core–shell structure and targeted delivery, polymeric micelles (PMs) is one of the most promising approaches for overcoming MDR and targeting to tumor site (Kedar et al., 2010). PMs are self-assembled from amphiphilic copolymers to form core–shell architecture. Since the hydrophilic shell minimizes protein adsorption on micelles and their cellular adhesion, PMs have the ability to evade no-specific capture by the mononuclear phagocytic system (MPS) (Li et al., 2015). Therefore, the nature of the hydrophilic shell impacts the characters of the micelles.

Currently, polyethylene glycol (PEG)-based block copolymer micelles and hyaluronic acid (HA)-based grafted copolymer micelles have been widely investigated in chemotherapy. HA, a natural glycosaminoglycan, has been extensively applied as “magic targeting ligand” for anticancer drug delivery (Yadav et al., 2007; Datir et al., 2012; Gao et al., 2015). HA-based micelles are beneficial in chemotherapy due to the specifically targeting to the CD44 receptors, which are over-expressed on the tumor surface (Almalik et al., 2013; Wang et al., 2016). However, HA-based micelles are limited by the MPS clearance and short blood circulation after intravenous administration (Kim et al., 2013; Han et al., 2015). PEG-based block copolymer micelles are used as vehicles for efficient drug delivery in chemotherapy, which make micelles stealth from host’s immune system, prolong blood circulatory time by reducing MPS clearance (Karlsson et al., 2005; Youk et al., 2005; Kamal et al., 2006; Kumar et al., 2009; Choi et al., 2011; Yin et al., 2017). Moreover, PEG-based micelles have smaller sizes but lower drug-loading capacity (Choudhury et al., 2017; Jin et al., 2017a, 2017b). Till now, little is known on which one is better between PEG-based micelles and HA-based micelles in chemotherapy drug delivery. In other words, which have more advantages in chemotherapy, EPR effect induced by PEG, or CD44-targeting effect induced by HA? Meanwhile, whether the mixed micelles with HA-based and PEG-based nanocarriers integrated the advantage of these two micelles, the mixed micelles are also seldom investigated in comparison with the corresponding HA-based micelles and PEG-based micelles.

Doxorubicin (DOX) is an anthracycline antibiotic as one of the most effective chemotherapy drugs in clinical cancer therapy, but it has been severely hindered for MDR and systemic toxic side-effects in clinical application (Pommier et al., 2010; Tacar et al., 2013). Curcumin (Cur) is an ideal chemosensitizer to downregulate MDR proteins, and inhibit cancer cell proliferation (Choi et al., 2008; Qian et al., 2011). Combination therapy of DOX with Cur could improve the anticancer effects and reduce the side-effects of DOX (Duan et al., 2012; Wang et al., 2013; Wang et al., 2015; Ma et al., 2017).

In this study, to study the differences among PEG-based block PM, HA-based grafted PMs and the mixed of these two micelles in anticancer drug delivery, two amphiphilic polymers with the same hydrophobic group of vitamin E succinate (VES) (HA-ethylenediamine-vitamin E succinate (HA-g-VES) and PEG 2000 VES (TPGS2k)), were applied as nanocarriers to establish HA-VES grafted PMs (HA-PMs), TPGS2k block PMs (TPGS2k-PMs) and a mixed micelles (HA/TPGS2k-PMs) for the co-delivery of DOX and Cur. The micelles were prepared and characterized. Meanwhile, the cytotoxicity, overcoming MDR efficiency, cellular uptake and endocytic mechanism of HA-PMs, TPGS2k-PMs and HA/TPGS2k-PMs were all investigated in MCF-7 and MCF-7/Adr cells. Finally, the antitumor activity and systemic toxicity *in vivo* were evaluated on 4T1 tumor-bearing mice.

## 2. Materials and methods

### 2.1. Materials

Doxorubicin hydrochloride (DOX·HCl) was purchased from Beijing Huafeng United Technology Co., Ltd. (Beijing, China). Cur was obtained from Sigma Aldrich (St. Louis, MO, USA). HA-g-VES (Wang et al., 2016) and PEG 2000 VES (TPGS2k) (Wang et al., 2012) was synthesized by our group. 3-(4,5-Dimethyl-2-thiazolyl)-2,5-diphenyl-2*H*-tetrazolium bromide (MTT) and dimethyl sulphoxide (DMSO) of analytical reagent grade were obtained from Sigma-Aldrich Co. (St. Louis, MO, USA). CK, CKMB, LDH and AST were obtained from Beijing Leadman Biochemistry Co. Ltd (Beijing, China). RMPI 1640 medium and fetal bovine serum (FBS) was provided by Gibco (BRL, MD, USA). All solvents used were of HPLC grade.

### 2.2. Animals

Female Balb/c mice weighing about 20 g were purchased from Beijing Vita River Company. All animal experiments were performed in accordance with guidelines for the Use and Care of Animals approved by the Beijing University of Chinese medicine Committee of Ethics. The animals were maintained at animal care facility with free access to standard food and water.

### 2.3. Preparation and characterization of HA/TPGS2k-PMs

HA/TPGS2k-PMs were prepared by a probe ultrasonication method as previous described (Ma et al., 2017). In brief, DOX·HCl was reacted with triethylamine to obtain DOX base. Then, 0.5 mL acetone solution containing 1 mg DOX and 1 mg Cur, was dripped into the PBS solution of 10 mg HA-g-VES and 10 mg TPGS2k. After 24 h stirring at room temperature, the mixture was sonicated at 200 W for 10 min under an ice bath by a probe-type ultrasonicator (Cole-Parmer, Vernon Hills Co. Ltd., IL, USA). The resulting micellar solution was filtered through 0.45 μm microfiltration membrane to remove unloaded drugs. The HA-PMs were prepared as the same procedure with 1 mg DOX and 1 mg Cur encapsulated into 20 mg HA-g-VES. Moreover, TPGS2k-PMs were prepared by thin-film hydration method with 1 mg DOX, 1 mg Cur and 40 mg TPGS2k (two times higher than the nanocarrier used in HA/TPGS2k-PMs and HA-PMs) (Wang et al., 2012).

The size distribution and zeta potential of micelles were determined by dynamic light scattering (DLS) with a Zetasizer (Nano ZS 90; Malvern Instruments Limited, Malvern Worces, UK). The morphology of HA/TPGS2k-PMs was visualized by transmission electron microscopy (TEM, Tecnai 20 200 kV, FEI, Hillsboro, USA) after negative stained with 1% uranyl acetate before observation.

The colloidal stability of HA/TPGS2k-PMs was evaluated to measure the changes of size and zeta potential after incubation with 1%, 5% and 10% FBS at 37 °C for 24 h. Meanwhile, the encapsulation efficiency (EE%) and drug-loading content (DL%) of DOX or Cur in micelles were analyzed by HPLC (Agilent 1260, Agilent Technologies, CA). The analysis was accomplished on a C18 reversed phase column (4.6 × 250 mm, 5 μm; Agilent Technologies) with the mobile phase of methanol-3 mmol/L monopotassium phosphate-acetic acid (58:42:0.5, v/v/v) with flow rate of 1 mL/min at 227 nm. The EE was calculated as the amount of total drug loaded in micelles to the amount of drug added in. The DL was also calculated as the amount of drugs loaded in micelles to the total mass of drug-loaded micelles. All the measurements were performed in triplicates.

The *in vitro* release profile of DOX or Cur from HA/TPGS2k-PMs was carried out by a dialysis method. Briefly, HA/TPGS2k-PMs were sealed in dialysis bags (MW 14,000), then the bags were immersed in 50 mL of phosphate buffer (pH 7.4, 6.5 and 5.5) or acetate buffer (pH 4.5) containing 0.5% Tween 80 (v/v), respectively, with a constant shaking of 100 rpm at 37 °C. At predetermined time intervals, 1 mL of release mediums were sampled and replenished by the same volume of fresh medium. The concentration of released DOX was analyzed by HPLC as described before. The *in vitro* release experiments were carried out in triplicate.

### 2.4. Cytotoxicity assay

The sensitive MCF-7 and multidrug-resistant MCF-7/Adr cells were obtained from Nanjing Kaiji Biotech. Ltd. Co. (Jiangsu, China). Cells were cultured in RPMI-1640 medium containing 10% (v/v) FBS and 1% penicillin–streptomycin at 37 °C with 5% CO_2_. Additionally, MCF-7/Adr cells were incubated in medium with 1 μg/mL DOX.

The cytotoxicity of various formulations against MCF-7 and MCF-7/Adr cells was assessed by MTT assay. Briefly, MCF-7 and MCF-7/Adr cells were seeded in 96-well plates at a density of 5 × 10^4^ cells/well/0.1 mL 1640 culture medium and cultured for 24 h. Then, the cells were treated with DOX-Sol, DOX + Cur, HA-PMs, TPGS2k-PMs and HA/TPGS2k-PMs with multiple concentrations of DOX for 48 and 72 h, respectively. The cells incubated with medium only were utilized as control. At time interval, 20 μL MTT (5 mg/mL) was added and incubated for another 4 h. After that, the medium of each well was discarded and added 200 μL DMSO. The absorbance of each well was measured at 570 nm using a microplate reader. Half-maximal inhibitory concentration (IC_50_), the resistant index (RI) and reversal factor (RF) were calculated and used to evaluate MDR reversal effects by micelles(Ling et al., 2010; Wang et al., 2012).

### 2.5. Cellular uptake in resistant MCF-7/Adr cells

The intracellular accumulation of DOX in resistant MCF-7/Adr cells was studied by confocal laser scanning microscopy (CLSM) and flow cytometry system (FCS). In the quantitative detection by FCS, MCF-7/Adr cells were grown in 24-well plates for 24 h, and then incubated with DOX-Sol, DOX + Cur, TPGS2k-PMs, HA-PMs and HA/TPGS2k-PMs at an equivalent DOX concentration. At the established time points, the cells were digested, collected, washed and acquired. The fluorescent intensity of DOX in cells was measured by FCS. In terms of CLSM visualization, DOX formations were incubated with MCF-7/Adr cells for 1 h and 2 h. Rinsed with cold PBS twice, fixed with 4% paraformaldehyde and counterstained to mark cell nucleus by 4′6-diamidino-2-phenylindole (DAPI). Cells were visualized by confocal laser scanning microscope (Olympus, Tokyo, Japan).

### 2.6. Cellular efflux in MCF-7/Adr cells

The efflux of various DOX formulations in MCF-7/Adr was investigated by FCS in MCF-7/Adr cells. Briefly, resistant MCF-7/Adr cells were incubated with DOX-Sol, DOX + Cur, TPGS2k-PMs, HA-PMs and HA/TPGS2k-PMs at an equivalent DOX concentration for 2 h. Then, the cells were washed with PBS and incubated with free medium for another 1 h and 2 h. After digestion and collection, the intracellular fluorescent intensity of DOX was measured by FCS.

### 2.7. Endocytosis pathway of HA/TPGS2k-PMs in MCF-7/Adr cells

The endocytosis pathway of HA/TPGS2k-PMs was investigated with specific endocytosis inhibitors by FCS in MCF-7/Adr cells. Initially, MCF-7/Adr cells were seeded in 24-well plates and cultured. After 24 h incubation, cells were pre-incubated with chlorpromazine (10 μg/mL), sodium azide (3 μg/mL), quercetin (6 μg/mL), indomethacin (6 μg/mL), amiloride (8 μg/mL), and β-cyclodextrin (1 mg/mL) for 1 h at 37 °C. Then, HA/TPGS2k-PMs and corresponding inhibitors were co-incubated with cells for another 1 h. After that, MCF-7/Adr cells were washed, digested, harvested, and detected by FCS.

### 2.8. In vivo pharmacokinetics

Male Sprague–Dawley rats were randomly assigned to five groups (five rats per group). DOX-Sol, DOX + Cur, TPGS2k-PMs, HA-PMs and HA/TPGS2k-PMs were injected intravenously with at the dose of 5 mg/kg DOX and 5 mg/kg Cur, respectively. About 300 μL of blood samples were collected from the orbital venous plexus and immediately centrifuged to obtain the plasma fraction. Plasma samples were extracted through methanol protein precipitation method before analyzed by LC–MS/MS system.

### 2.9. In vivo biodistribution

The *in vivo* biodistribution and tumor imagination of DOX-Sol, DOX + Cur, TPGS2k-PMs, HA-PMs and HA/TPGS2k-PMs were evaluated in 4T1-bearing Balb/C mice quantitatively and qualitatively. Briefly, different DOX formulations were intravenously injected via tail vein into 4T1-bearing Balb/C mice at the dose of 10 mg DOX/kg and 10 mg Cur/kg, respectively. After 2 h administration, the mice were sacrificed, and the tumor, heart, liver, lung, and kidney were excised, weighed, homogenized in 0.5 mL methanol, centrifuged and analyzed by LC–MS/MS. Meanwhile, for qualitative analysis, the tumors were frozen in cryoembedding media (OCT) and sectioned at 20 µm. The sections were fixed with 4% paraformaldehyde for 10 min, stained for nuclei with DAPI, and observed by CLSM.

### 2.10. In vivo antitumor efficacy and safety

The antitumor efficacy and safety *in vivo* were evaluated using 4T1 tumor-bearing mice. Briefly, 1 × 10^6^ 4T1 cells were subcutaneously injected into the right axilla of the female BALB/c mice. When the tumors reached about 100 mm^3^, the mice were randomly divided into five groups (10 mice per group), and intravenously injected with saline (as control) and equal dose of 10 mg/kg DOX with DOX-Sol, DOX + Cur, TPGS2k-PMs, HA-PMs and HA/TPGS2k-PMs every other two days. Body weights and tumor volumes (*V* = *ab*^2^/2, where *a* was major axis and *b* was minor axis measured by slide caliper) were measured every two days after administration. At the end of the experiment, mice were sacrificed, and then the tumors were excised, weighted and photographed.

The toxicity of DOX formulations *in vivo* were evaluated by enzyme-linked immunosorbent assay (ELISA) kit. At the end of the experiment, the blood of each mouse was extracted by eyeballs and the serum was separated. Then, the activities of creatine kinase (CK), creatine kinase MB (CKMB), lactate dehydrogenase (LDH) and aspartate transaminase (AST) in serum were evaluated to investigate the organ toxicity.

### 2.11. Statistical analysis

Results were expressed as mean ± SD (standard deviation). A student’s *t*-test or one-way ANOVA was applied to test for significance in the experiments. Statistical differences were considered significant at *p* < .05 and very significant at *p* < .01.

## 3. Results and discussion

### 3.1. Characterization of HA/TPGS2k-PMs

**Figure 1.**
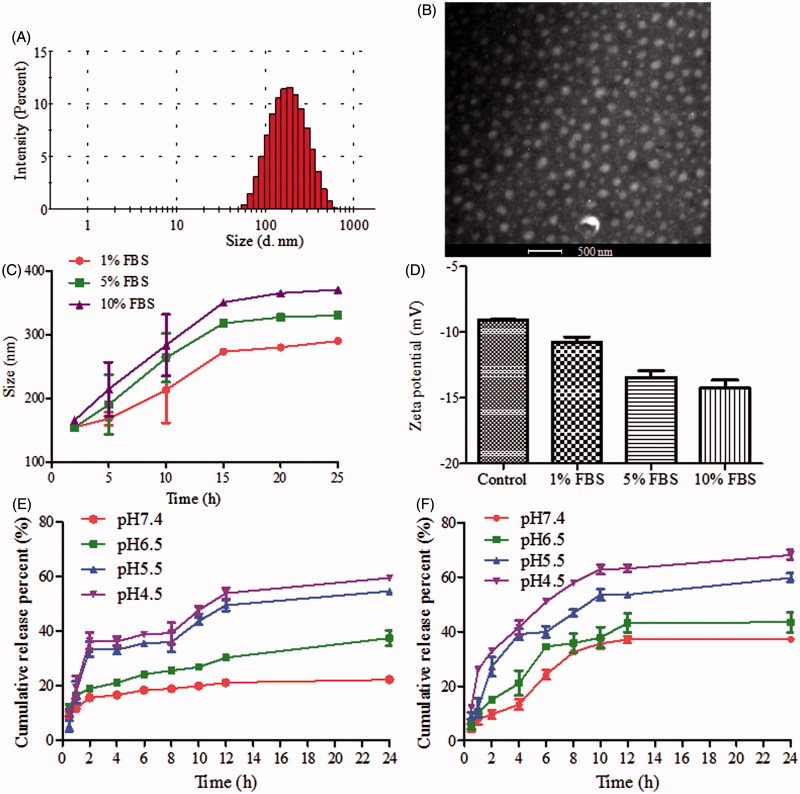
(A) Size distribution and (B) TEM image of HA/TPGS2k-PMs. (C) Size and (D) zeta potential changes of HA/TPGS2k-PMs in 1%, 5% and 10% BSA aqueous at 37 °C for 24 h. (E and F) *In vitro* cumulative release profiles of (E) DOX and (F) Cur from HA/TPGS2k-PMs at different pH values (mean ± SD, *n* = 3).

HA/TPGS2k-PMs were prepared the same as HA-PMs by a probe ultrasonication method with DOX and Cur co-encapsulated into the hydrophobic inner core of HA-g-VES and TPGS2k, and the total amount of nanocarriers in HA/TPGS2k-PMs was the same as that in HA-PMs. However, TPGS2k-PMs were prepared by thin-film hydration. In order to achieve the similar drug concentration and encapsulation efficiency, the amount of polymer in TPGS2k-PMs was two times higher than that in HA-PMs and HA/TPGS2k-PMs. It indicated that the loading activity of HA-g-VES was higher than that of TPGS2k. As shown in Table S1 and Figure 1, the particle size of HA/TPGS2k-PMs was 153.37 ± 1.00 nm, which was smaller than that of HA-PMs (223.83 ± 1.84), revealing that the micelles were more tight due to the addition of TPGS2k. Moreover, in comparison with the zeta potential of HA-PMs (−10.43 mV), HA/TPGS2k-PMs had a zeta potential of -9.43 mV, which was due to the neutral PEG on the micellar surface and beneficial for reducing the MPS-mediated clearance. But the zeta potentials between them have no significant differences. The DL in HA/TPGS2k-PMs was 7.10 ± 0.32%, which was lower than that in HA-PMs (8.31 ± 0.15%) but higher than TPGS2k-PMs (4.38 ± 0.24%). It confirmed that HA-based grafted copolymer micelles had higher-loading capacity than PEG-based block copolymer micelles. Overall, HA graft copolymer and PEGylated block copolymer have their own features, specifically, HA-based micelles have a high drug-loading capacity with a little large particle size of 200 nm, but PEGylated micelles have a small size of 20 nm with relative low drug-loading capacity.

The colloidal stability of HA/TPGS2k-PMs was evaluated in different FBS aqueous (Figure 1(C,D)), which was nearly the same as that of HA-PMs and TPGS2k-PMs (Wang et al., 2016). The particle size of HA/TPGS2k-PMs was increased with the augment of incubation time and the concentration of FBS. The particle size of micelles increased to 1.9-fold of the original size after 24 h in 5% FBS (the physiological concentration of FBS) aqueous, but with good dispersibility. When the concentration of FBS increased to 10%, the micelles were 2.3-fold increase in particle size, but with no visible precipitation. That might be mainly due to the adsorption of FBS onto the surface of the micelles, which led to the micellar swelling, increasing particle size, and the decreased stability of micelles with the increasing FBS concentration. The zeta potentials of HA/TPGS2k declined slightly because of the negative charge of FBS, but HA/TPGS2k were relatively stable with no precipitation.

The release profiles of DOX and Cur from HA/TPGS2k-PMs were shown in Figure 1(E,F). The release of DOX and Cur from HA/TPGS2k-PMs was pH-dependent, which was fast in pH 4.5 and pH 5.5, but slow in pH 6.5 and pH 7.4. It indicated that drugs could be released from HA/TPGS2k-PMs more rapidly in endosome and lysosome, and would have more effective antitumor effects on tumor cells. Moreover, as our previous results (Wang et al., 2015), the release behaviors of HA/TPGS2k-PMs were similar with that of HA-PMs, which suggested that HA-PMs with or without TPGS2k have similar stability. However, when the micelles of TPGS2k-PMs and HA/TPGS2k-PMs have the similar stability and release behaviors, the weight ratio of nanocarrier and drug in HA-PMs and TPGS2k-PMs was 10:1 and 20:1 (w/w), respectively. Overall, HA-based micelles enhance the drug-loading capacity and stability in comparison with PEG-based micelles.

### 3.2. Cytotoxicity assay

**Figure 2.**
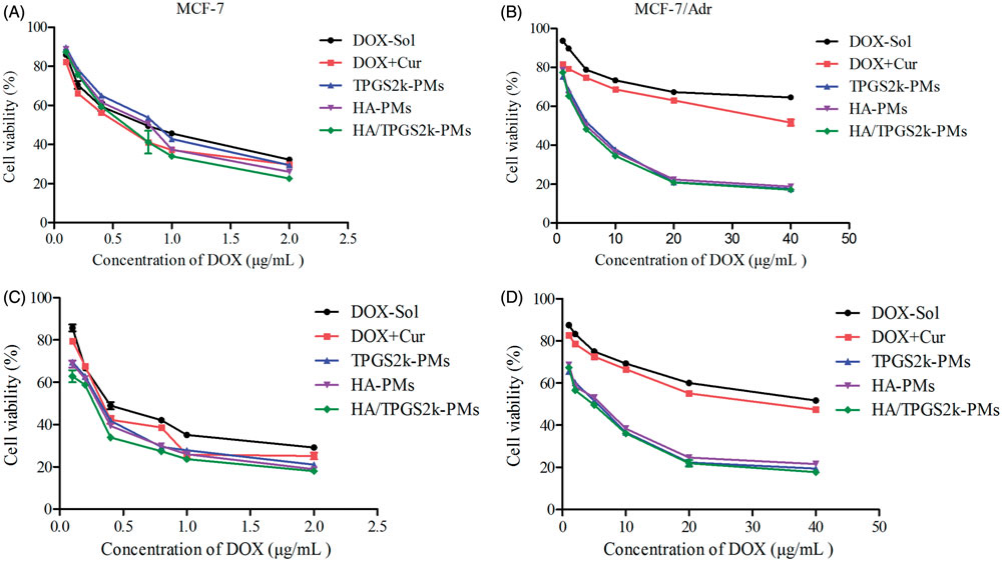
*In vitro* cytotoxicity of different DOX formulations against MCF-7 (A and C) and MCF-7/Adr cells (B and D) at 48 and 72 h post-treatment, respectively (mean ± SD, *n* = 3).

The cytotoxicity of DOX formulations was evaluated in MCF-7 and MCF-7/Adr cells by MTT. As shown in Figure 2 and Table S2, after 48 h incubation the IC_50_ value of DOX was 106.50 µg/mL in MCF-7/Adr cells, which was 142-fold resistant to that in MCF-7 cells (0.75 µg/mL). The addition of Cur significantly reduced the IC_50_ values of DOX, and the RI was decreased to 113.0, implying the synergistic antitumor effect of Cur on DOX (Table S3). Specifically, the IC_50_ values of drugs-loaded micelles were much lower than that of DOX + Cur, which demonstrated that co-loaded micelles were more effective in reducing cellular surviving and inducing anticancer activity in comparison with free DOX + Cur and DOX in resistant MCF-7/Adr cells. Moreover, TPGS2k-PMs have a higher IC_50_ value in MCF-7 cells in comparison with HA-PMs, because of the CD44 targeting activity by HA-PMs in MCF-7 cells (CD44-high expressed). However, due to the nano-size and the P-gp inhibitory effect by PEG-based micelles, the IC_50_ and RF values of TPGS2k-PMs were lower in MCF-7/Adr cells. Among these three micelles, the IC_50_ value of HA/TPGS2k-PMs in MCF-7 and MCF-7/Adr cells was the lowest, indicating that HA/TPGS2k-PMs combined the advantage of HA-based micelles and PEG-based micelles and achieved the highest cytotoxicity. Above all, the results suggested that HA-based micelles have CD44 targeting activity in MCF-7 cells, and PEG-based micelles exhibited strong cytotoxicity and reversal MDR effect in MCF-7/Adr cells, but the mixed micelles of HA/TPGS2k integrated the advantages of these two micelles and could significantly improve cytotoxicity and better overcome MDR than HA-PMs and TPGS2k-PMs *in vitro*.

### 3.3. Cellular uptake and efflux

**Figure 3.**
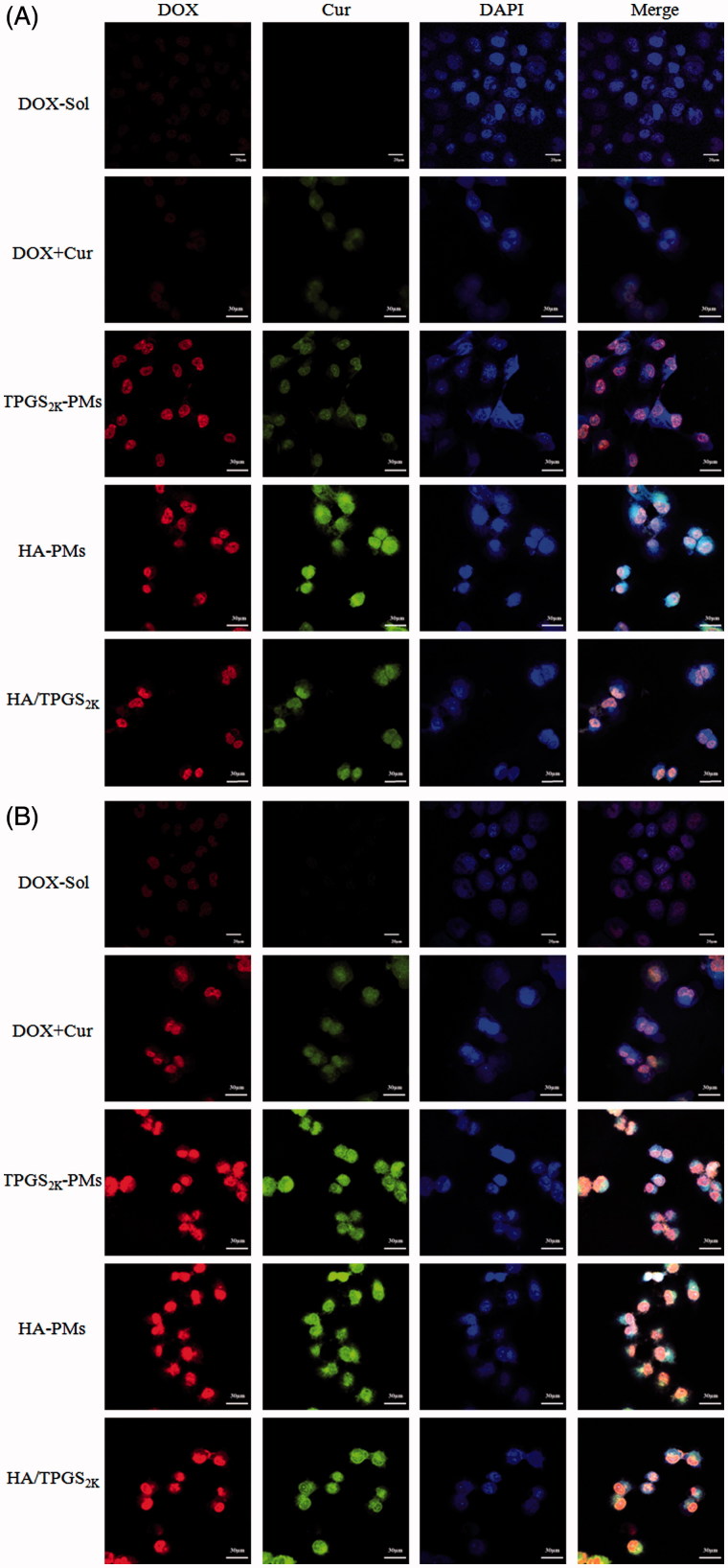
The CLSM images of DOX, DOX + Cur, HA-PMs, TPGS2k-PMs and HA/TPGS2k-PMs in MCF-7/Adr cells after incubation with 1 h (A) and 2 h (B). Cells were counterstained with DAPI for nuclei. Scale bar: 30 μm.

The cellular uptake of various DOX formulations was investigated in MCF-7/Adr cells by CLSM and FCS. As the qualitative CLSM images shown in Figure 3, Cur enhanced the cellular uptake of DOX in MCF-7/Adr cells. Meanwhile, HA-PMs, TPGS2k-PMs and HA/TPGS2k-PMs obviously promoted the internalization and accumulation of DOX in the nucleus of MCF-7/Adr cells compared with free DOX + Cur in a time-dependent manner. Moreover, the enhanced cellular uptake of co-loaded micelles was further confirmed by FCS in MCF-7/Adr cells. As shown in Figure S1, the cellular uptake of DOX in three co-loaded micelles was much faster and more effective than that in free DOX and even in DOX + Cur. These three micelles exhibited similar absorption promoting effects on DOX after 2 h incubation. Effectively, after incubation 4 h in MCF-7/Adr cells, more DOX was taken in TPGS2k-PMs in comparison with HA-PMs, but the cellular uptake of DOX in HA/TPGS2k-PMs was the highest.

Excitingly, as the cellular efflux results shown in Figure S1 B, Cur obviously inhibited the efflux of DOX due to P-gp inhibition. Co-loaded micelles were all significantly decreased the efflux of DOX in comparison with DOX + Cur. The efflux inhibitory activity of DOX in three co-loaded micelles had no significant differences at 2 h. After incubation 4 h, HA/TPGS2k-PMs and TPGS2k-PMs exerted similar efflux inhibition, and these two had higher fluorescence intensity than HA-PMs. Overall, three co-loaded micelles not only obviously increased the cellular uptake of DOX, but also decreased the efflux of DOX. Meanwhile, the cellular uptake of DOX in MCF-7/Adr cells was higher in TPGS2k-PMs than that in HA-PMs, and HA/TPGS2k-PMs displayed the best uptake promotion and efflux inhibition in these three micelles. Therefore, nano-size (about 20 nm), P-gp inhibition by PEGylated micelles exerted a better ability of improved cytotoxicity, promoting cellular uptake and reversal MDR of DOX than CD44-targeting effects by HA-based micelles in MCF-7/Adr cells *in vitro*. Excitingly, due to the hybrid effects of reversal MDR by PEG and CD44 targeting by HA, the mixed HA/TPGS2k-PMs among the three micelles ultimately had a better cytotoxicity and reversal MDR effect *in vitro*.

### 3.4. Endocytosis mechanism

The endocytic mechanism of HA/TPGS2k-PMs was investigated by the addition of endocytosis inhibitor (Bareford & Swaan, 2007; Sahay et al., 2010; He et al., 2013). As the results shown in Figure S1 C, quercetin (a caveolae- and clathrin-independent endocytosis inhibitor), indometacin (cyclooxygenase and caveolae-mediated endocytosis inhibitor) and β-cyclodextrin (an inhibitor of lipid raft and caveolae-dependent endocytosis) had no effects on the cellular uptake of HA/TPGS2k-PMs, indicating that caveolae- and clathrin-independent endocytosis and caveolae-dependent endocytosis were not involved in the endocytosis of the mixed micelles. However, chlorpromazine (a clathrin-mediated endocytosis inhibitor), sodium azide (energy inhibitor) (*p* < .01) and amiloride (Na^+^/H^+^ pump and micropinocytosis inhibitor) (*p* < .05) decreased the cellular uptake of DOX, proving that the endocytosis mechanism of HA/TPGS2k-PMs was micropinocytosis, energy-dependent and clathrin-mediated endocytosis.

Moreover, the endocytosis mechanism of HA-PMs was energy-dependent and caveolae-mediated endocytosis, and TPGS2k-PMs internalized by energy-dependent, caveolae-dependent endocytosis, and caveolae- and clathrin-independent endocytosis. Therefore, the endocytosis mechanism of HA/TPGS2k-PMs was different from that of HA-PMs and TPGS2k-PMs.

### 3.5. Pharmacokinetics in vivo

The mean plasma concentration–time profiles of different DOX formulations were shown in Figure S2. And the main pharmacokinetic parameters were calculated using non-compartmental model (Tables S4 and S5). Cur increased the plasma concentration of DOX *in vivo* in comparison with DOX-Sol, due to the absorption improvement of Cur. As expected, HA-PMs, TPGS2k-PMs and HA/TPGS2k-PMs all showed increased concentration of DOX and Cur in plasma compared with those of DOX + Cur (shown in Figure S2). It once again proved that micelles could significantly improve the absorption of encapsulated drugs. As shown in Table S4, *t*_1/2_ and AUC_(0–t)_ of DOX in TPGS2k-PMs was significantly higher than that in HA-PMs (*p* < .01), but those were no significant difference with HA/TPGS2k-PMs. This result indicated that in comparison with HA-based micelles, PEGylated micelles could not only extended the blood circulation time, but also enhance drug absorption *in vivo*. Similarly, main pharmacokinetic parameters of AUC_(0–t)_ and *t*_1/2_ of Cur in TPGS2k-PMs were similar with those in HA/TPGS2k-PMs, but some more than those in HA-PMs (*p* < .05) (Table S5). In conclusion, pharmacokinetics *in vivo* proved that due to the prolonging the blood circulation by PEG, PEGylated micelles could improve plasma concentration to a certain extent.

### 3.6. Biodistribution in vivo

**Figure 4.**
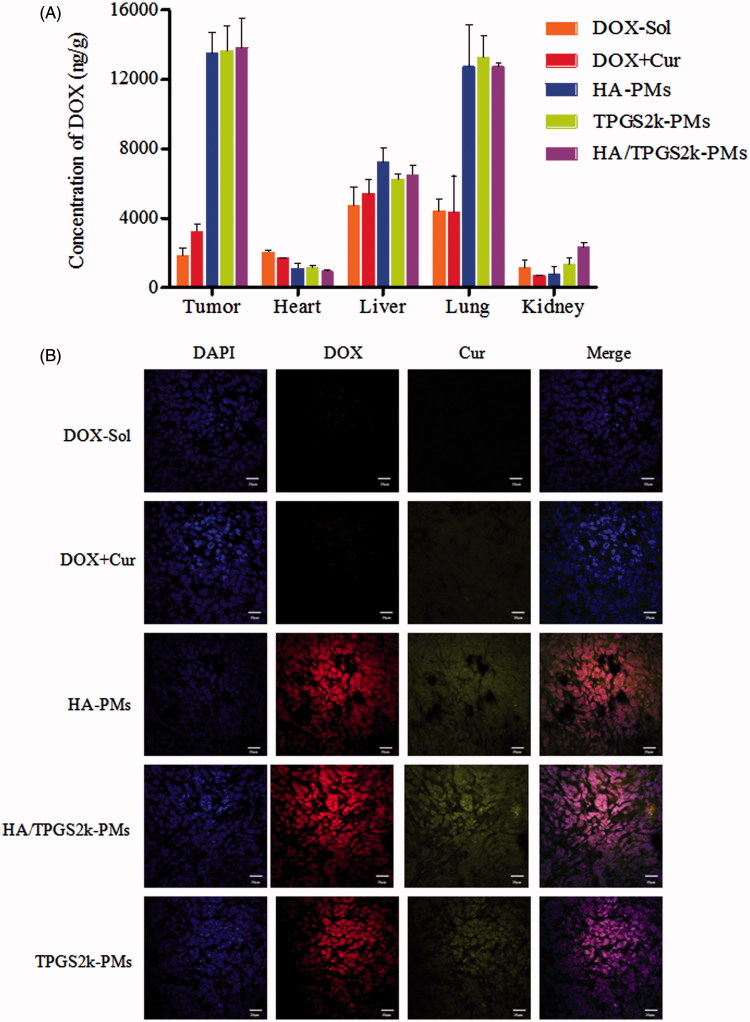
(A) Biodistribution of DOX in 4T1 tumor-bearing mice after 2 h intravenous administration with DOX-Sol, DOX + Cur, HA-PMs, TPGS2k-PMs and HA/TPGS2k-PMs. (B) CLSM images of frozen section of tumor separated from 4T1 tumor-bearing mice after 2 h intravenous administration with different formulations (scale bar: 20 µm).

The biodistribution of DOX-Sol, DOX + Cur, HA-PMs, TPGS2k-PMs and HA/TPGS2k-PMs in 4T1 tumor-bearing mice after 2 h administration was quantitatively evaluated in major organs including tumor, heart, liver, lung, and kidney. Initially, DOX-Sol and DOX + Cur treated groups displayed low DOX accumulation and no obvious differences in tumor *in vivo* (shown in Figure 4(A)). HA-PMs, TPGS2k-PMs and HA/TPGS2k-PMs all significantly enhanced tumor accumulation, which would be beneficial to their antitumor effect *in vivo*. However, the amount of three micelles in tumor showed no significant variances, indicating that the antitumor effect of pegylation improving blood circulation time and reducing RES capture was equal to that of hyalacylation targeting CD44 receptor in tumor. However, the quantitative analysis of Dox in liver, HA/TPGS2k-PMs and TPGS2k-PMs were relative low concentration compared with HA-PMs, demonstrating that pegylated modification could reduce the recognization by HARE receptor in liver and the capture of RES (Han et al., 2015; Yang et al., 2018). HA-PMs, HA/TPGS2k-PMs and TPGS2k-PMs exhibited lower heart accumulation in comparison with DOX-Sol and even DOX + Cur, but with no significant differences among them, indicating that drugs encapsulated into micelles could decrease the cardiotoxicity. However, TPGS2k-PMs and HA/TPGS2k-PMs exhibited higher uptake by kidney in comparison with HA-PMs *in vivo*, and three micelles all exhibited a high accumulation in lung compared with DOX-Sol.

As shown in Figure 4(B), tumor tissues were frozen, sectioned, stained with DAPI, and imagined under CLSM. As we can see from the CLSM images, drugs were all internalized into the cell nuclear. The red fluorescence of DOX and green fluorescence of Cur were significantly increased in HA-PMs, TPGS2k-PMs and HA/TPGS2k-PMs treated groups compared with DOX-Sol and DOX + Cur treated groups, which visually showed that the micelles could increase tumor permeability and tumor targeting. Meanwhile, the fluorescence of DOX and Cur was no significant difference in these three micelles. Therefore, the CLSM results were consistent with the quantitative results of the distribution in tumor and it further confirmed that PEGylated micelles with EPR passive targeting effects have no significantly differences with the HA-based micelles with CD44 active targeting effects.

### 3.7. Antitumor effects in vivo

**Figure 5.**
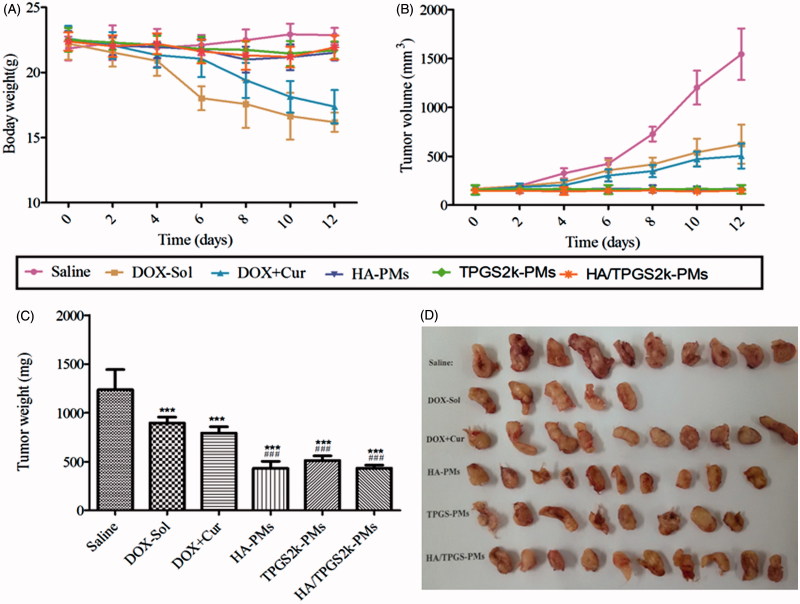
*In vivo* antitumor effects on 4T1-bearing mice after intravenous administration with saline, DOX, DOX + Cur, HA-PMs, TPGS2k-PMs and HA/TPGS2k-PMs. The changes of mice weight (A) and tumor volume (B) after treated with different formulations at a dose of 10 mg DOX/kg. The tumor weight (C) and representative excised tumor (D) from the sacrificed mice at day 10 (**p < .01 compared with saline group, ###*p* < .001 compared with DOX group).

Tumor suppression *in vivo* was analyzed to evaluate the synergistic antitumor activity of three DOX + Cur co-loaded micelles. As shown in Figure 5(A), the body weight of the saline-treated mice was increased due to the fast growth of tumor. DOX caused serious side effects, which lead the body weight markedly decreased. Moreover, the body weight of the DOX + Cur treated mice was decreased, but that was seriously higher than DOX-treated mice, proving that Cur could decrease the side effects of DOX. Interestingly, three co-loaded micelles significantly improved the survival quality of tumor-bearing mice, but the body weight of three DOX + Cur co-loaded micelles had no significant variation. Excitingly, three co-loaded micelles decreased tumor volume and tumor weight, and exhibited the strongest anti-tumor activity with no differences (Figure 5(B,C)). Therefore, the synergistic antitumor activity of HA-PMs, TPGS2k-PMs and HA/TPGS2k-PMs not only significantly increased tumor suppression, but also decreased the side effects of DOX in comparison with DOX or even DOX + Cur.

As shown in Figure 5(D), there are only five tumors in DOX-Sol treated group, because five mice suddenly died after nine days treatment of DOX-Sol, which indicated the serious side effects of DOX-Sol. However, although the tumor weight and volume in DOX + Cur treated mice was similar with DOX-Sol treated mice, the mice were in good state without death after 12 days treatment with DOX + Cur, which proved once again that Cur decreased the side effects of DOX. The size of the collected tumor was significantly decreased in the mice treated with HA-PMs, TPGS2k-PMs and HA/TPGS2k-PMs, in accordance with the above results. However, these three co-loaded micelles displayed no difference in antitumor effects *in vivo*, demonstrating that the PEG passive and HA active targeted delivery micelles had no significant variances *in vivo*. Also, it indicated that the PEG-based micelles with nano-size (lower than 50 nm) leading EPR effects had the similar antitumor effect with the HA-based micelles owing CD44 targeted effect and larger size above 150 nm. Furthermore, to better increase the antitumor effect, the micelles should self-assemble with active target PEGylated nanocarrier or PEGylated HA nanocarrier, in other words, the micelles should have nano-size and active target effects.

**Figure 6.**
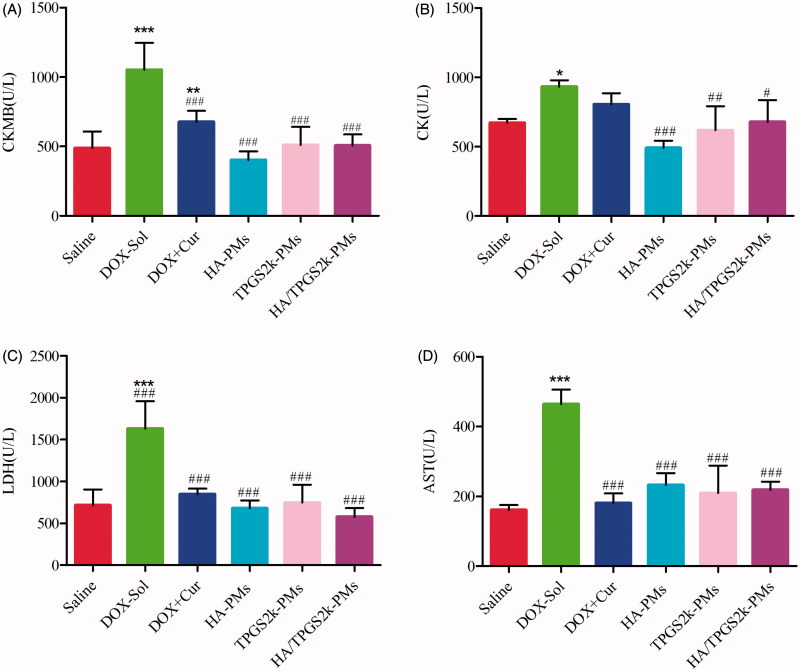
Evaluation the levels of (A) CKMB, (B) CK, (C) LDH, and (D) AST after intravenous administration with saline, DOX, DOX + Cur, HA-PMs, TPGS2k-PMs and HA/TPGS2k-PMs in 4T1-bearing mice. (*p < .05, **p < .01, ***p < .001 compared with saline group, #*p* < .05, ##*p* < .01, ###*p* < .001 compared with DOX-Sol group).

The activities of CK, CKMB, LDH and AST in serum were evaluated to investigate the toxicity of different DOX formulations. As shown in Figure 6, the values of these four parameters in DOX-Sol treated groups were all significantly higher than those in saline-treated groups, demonstrating the high toxicity of DOX-Sol. The values of CKMB, CK, LDH and AST in the groups treated with (DOX + Cur)-Sol, HA-PMs, TPGS2k-PMs and HA/TPGS2k-PMs were all significantly decreased compared with those in DOX-Sol treated group. It could indicate that Cur solution or mixed micelles all reduced the cytotoxicity including cardiotoxicity, hepatotoxicity or so on caused by DOX. Overall, DOX and Cur co-loaded micelles could not only significantly enhance the antitumor effects, but also reduced the side effects of DOX *in vivo* with no differences. Thereafter, HA-g-VES graft copolymer micelles and PEG-VES (TPGS2k) block copolymer micelles have different advantages, but showed similar antitumor effects after i.v. injection. We concluded that the passive targeting effects of EPR caused by PEG and the active targeting effects of CD44 caused by HA have no significantly variance in the delivery of antitumor drugs by i.v.

## 4. Conclusions

In this study, two amphiphilic polymers of HA-g-VES and TPGS2k with the same hydrophobic group of VES were used as carriers for the co-delivery of DOX and Cur. And the differences among HA-based grafted polymer micelles, PEG-based block polymer micelles, and mixed of these two micelles in enhancing antitumor effects and overcoming MDR were also evaluated. The loading capacity of HA-PMs and HA/TPGS2k-PMs was two times higher than TPGS2k-PMs, but TPGS2k-PMs have a smaller size (13.21 nm) than HA-PMs (223.93 nm) and HA/TPGS2k-PMs (153.37 nm). Moreover, TPGS2k-PMs enhanced the cytotoxicity and MDR reversal effect on MCF-7/Adr cells in comparison with HA-PMs. On the account of the hybrid effects of nano-size and CD44 targeting efficiency, HA/TPGS2k-PMs ultimately exhibited superior cytotoxicity, intracellular accumulation and reversal MDR effect among these three micelles *in vitro*. Finally, the *in vivo* antitumor study exhibited that three DOX + Cur co-loaded micelles significantly improved the antitumor effects and reduced the size effects of DOX with no differences. Therefore, it concluded that the EPR passive targeting effects caused by PEGylated micelles and the CD44 active targeting effects caused by HA-based micelles have no significantly variances in the delivery of antitumor drugs by i.v.

## Supplementary Material

IDRD_Wang_et_al_Supplemental_Content.docx
