# Supplementary material for: Comparison of hyaluronic acid-based micelles and polyethylene glycol-based micelles on reversal of multidrug resistance and enhanced anticancer efficacy in vitro and in vivo
Source: Drug Deliv. 2018 Jan 19;25(1):330–40. doi: 10.1080/10717544.2018.1428385 (PMC6058673; doi:10.1080/10717544.2018.1428385)
Supplement: IDRD_Wang_et_al_Supplemental_Content.docx [file IDRD_A_1428385_SM6053.docx]

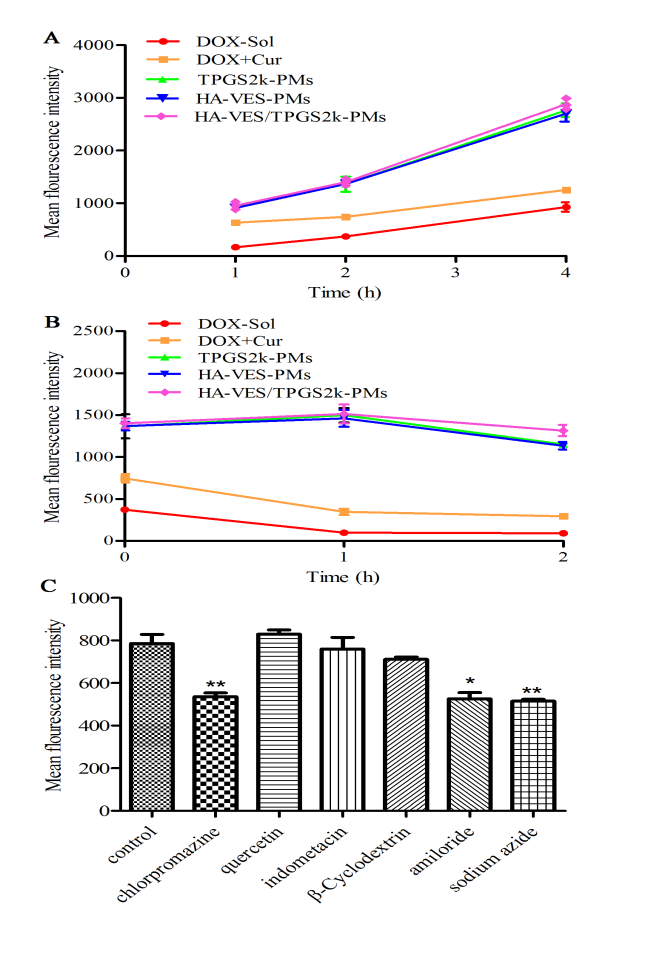


Fig.S1 (A) Cellular uptake of DOX in MCF-7/Adr cells after incubation with DOX, DOX+Cur, HA-PMs, TPGS2k-PMs and HA/TPGS2k-PMs for 1 h, 2 h, and 4 h, respectively. (B) The efflux of DOX from MCF7/Adr cells after initial incubation with different DOX formulations for 2 h and then replaced with fresh medium for another 1 and 2 h incubation. (C) Endocytosis mechanism of HA/TPGS2k-PMs in MCF7/Adr cells after incubation with different endocytosis inhibitors determined by FCS. (n=3, mean ± SD, *p<0.05, **p<0.01 vs control).


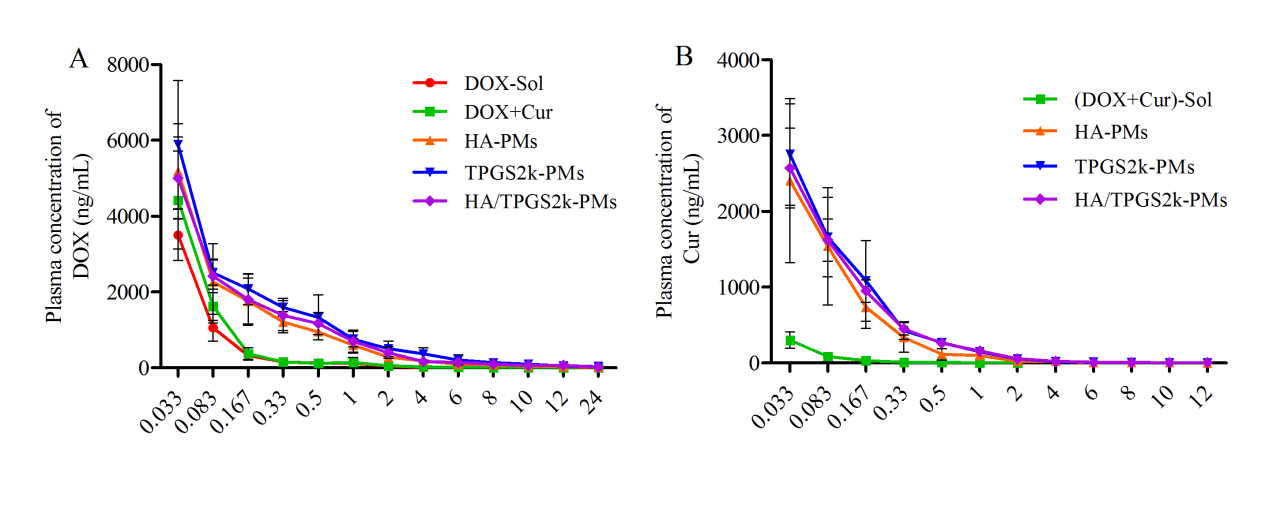


Fig.S2 Plasma concentration-time profiles of DOX (A) and Cur (B) in rats after intravenous administration with different DOX formulations at a dose of 5 mg·kg^-1^ DOX and 5mg·kg^-1^ Cur. (n=5, Mean ± SD)

Table S1 The physical characterizations of HA-PMs, TPGS2k-PMs and HA/TPGS2k-PMs (mean±SD, n=3).

|  | Size (nm) | PDI | Zeta potential (mV) | EE (%) | | DL (%) |
| --- | --- | --- | --- | --- | --- | --- |
|  |  |  |  | DOX | CUR |  |
| HA/TPGS2k | 153.37±1.00 | 0.23±0.01 | -9.43±0.68 | 88.20±0.51 | 68.05±0.53 | 7.10±0.32 |
| HA-PMs | 223.83±1.84 | 0.27±0.01 | -10.43±0.52 | 94.82±0.62 | 72.56±0.48 | 8.31±0.15 |
| TPGS2k-PMs | 13.21 ± 0.13 | 0.20±0.02 | 0.41 ± 0.15 | 96.28±0.28 % | 89.32±0.22 % | 4.38±0.24 |

Table S2 IC_50_ values of DOX-Sol, DOX+Cur, HA-PMs, TPGS2k-PMs and HA/TPGS2k-PMs in MCF-7 and MCF-7/Adr cells after 48 and 72 h incubation.

| Frmulations | IC_50_ (µg/mL) | | | | |
| --- | --- | --- | --- | --- | --- |
|  | MCF-7 | |  | MCF-7/Adr | |
|  | 48h | 72h |  | 48h | 72h |
| DOX | 0.75 | 0.51 |  | 106.50 | 45.81 |
| DOX+Cur | 0.55 | 0.40 |  | 62.15 | 34.34 |
| TPGS2k | 0.81 | 0.31 |  | 4.99 | 3.83 |
| HA-PMs | 0.69 | 0.29 |  | 4.92 | 4.21 |
| HA/TPGS2k | 0.58 | 0.23 |  | 4.50 | 3.55 |

Table S3 The resistant index (RI) and the reversal factor (RF) of different formulations at 48 and 72 h in MCF-7/Adr cells.

| Formulations | RI | |  | RF | |
| --- | --- | --- | --- | --- | --- |
|  | 48h | 72h |  | 48h | 72h |
| DOX | 142.01 | 89.82 |  | – | – |
| DOX+Cur | 113.0 | 85.85 |  | 1.71 | 1.33 |
| TPGS2k | 6.16 | 12.35 |  | 21.34 | 11.96 |
| HA-PMs | 7.13 | 14.52 |  | 21.65 | 10.88 |
| HA/TPGS2k | 7.76 | 15.43 |  | 23.67 | 12.90 |

Table S4 Pharmacokinetic parameters of DOX after intravenous administration of DOX-Sol, DOX+Cur, HA-PMs, TPGS2k-PMs and HA/TPGS2k-PMs at a dose of DOX 5 mg ·kg^-1^ and Cur 5 mg·kg^-1^.

| Parameter |  | DOX-Sol | DOX+Cur | HA/TPGS2k-PMs | HA-PMs | TPGS2k-PMs |
| --- | --- | --- | --- | --- | --- | --- |
| AUC(0-t) | µg/L*h | 616.60±95.52 | 897.47±83.67 | 3599.82 ±601.28 | 3169.64±445.35 | 3951.26±543.92 |
| AUC(0-∞) | µg/L*h | 761.13±283.71 | 914.79±91.60 | 3711.56± 408.32 | 3327.38± 473.52 | 4325.54±625.26 |
| MRT(0-t) | h | 0.93±0.38 | 2.13±1.61 | 3.98±0.57 | 3.85±0.83 | 4.25±0.52 |
| MRT(0-∞) | h | 1.68±0.98 | 2.48±1.77 | 5.68±2.13 | 5.36±1.81 | 6.38±0.65 |
| t_1/2_ | h | 2.40±1.79 | 3.79±2.33 | 7.35±0.84 | 6.94±1.15 | 7.68±1.08 |
| T_max_ | h | 0.033±0.00 | 0.033±0.00 | 0.033±0.00 | 0.033±0.00 | 0.033±0.00 |
| CL | L/h/kg | 0.007±0.00 | 0.006±0.00 | 0.003±0.00 | 0.002±0.00 | 0.00 |
| V | L/kg | 0.03±0.02 | 0.03±0.02 | 0.03±0.02 | 0.03±0.01 | 0.04±0.02 |
| C_max_ | µg/L | 3504.±676.34 | 4353.33±1168.89 | 5320±1842.31 | 5188.00±1246.00 | 5921.66±1958.45 |

Table S5 Pharmacokinetic parameters of Cur after intravenous administration of DOX+Cur, HA-PMs, TPGS2k-PMs and HA/TPGS2k-PMs at a dose of DOX 5 mg ·kg^-1^ and Cur 5 mg·kg^-1^.

| Parameter |  | DOX+Cur | HA/TPGS2k-PMs | HA-PMs | TPGS2k-PMs |
| --- | --- | --- | --- | --- | --- |
| AUC(0-t) | µg/L*h | 92.26±57.39 | 586.12±205.65 | 550.45±306.72 | 612.54±203.46 |
| AUC(0-∞) | µg/L*h | 97.18± 66.92 | 603.89±305.04 | 565.52±303.18 | 623.48±218.08 |
| MRT(0-t) | h | 0.39±0.22 | 1.31±0.21 | 1.24±0.18 | 1.49±0.45 |
| MRT(0-∞) | h | 0.45±0.29 | 2.01±0.24 | 1.88±0.95 | 2.38±0.61 |
| t_1/2_ | h | 0.27±0.24 | 4.20±1.45 | 3.72±2.23 | 4.60±1.95 |
| t _max_ | h | 0.033±0.00 | 0.033±0.00 | 0.033±0.00 | 0.033±0.00 |
| CL | L/h/kg | 0.07±0.04 | 0.05±0.01 | 0.01±0.00 | 0.06±0.01 |
| V | L/kg | 0.02±0.00 | 0.06±0.08 | 0.06±0.05 | 0.07±0.06 |
| C _max_ | µg/L | 455.00±110.48 | 2643.01±5551.01 | 2468.60±1193.85 | 2750.42±668.69 |
